# Nengo: a Python tool for building large-scale functional brain models

**DOI:** 10.3389/fninf.2013.00048

**Published:** 2014-01-06

**Authors:** Trevor Bekolay, James Bergstra, Eric Hunsberger, Travis DeWolf, Terrence C. Stewart, Daniel Rasmussen, Xuan Choo, Aaron Russell Voelker, Chris Eliasmith

**Affiliations:** Centre for Theoretical Neuroscience, University of WaterlooWaterloo, ON, Canada

**Keywords:** neural engineering framework, nengo, Python, neuroscience, theoretical neuroscience, control theory, simulation

## Abstract

Neuroscience currently lacks a comprehensive theory of how cognitive processes can be implemented in a biological substrate. The Neural Engineering Framework (NEF) proposes one such theory, but has not yet gathered significant empirical support, partly due to the technical challenge of building and simulating large-scale models with the NEF. Nengo is a software tool that can be used to build and simulate large-scale models based on the NEF; currently, it is the primary resource for both teaching how the NEF is used, and for doing research that generates specific NEF models to explain experimental data. Nengo 1.4, which was implemented in Java, was used to create Spaun, the world's largest functional brain model (Eliasmith et al., 2012). Simulating Spaun highlighted limitations in Nengo 1.4's ability to support model construction with simple syntax, to simulate large models quickly, and to collect large amounts of data for subsequent analysis. This paper describes Nengo 2.0, which is implemented in Python and overcomes these limitations. It uses simple and extendable syntax, simulates a benchmark model on the scale of Spaun 50 times faster than Nengo 1.4, and has a flexible mechanism for collecting simulation results.

## 1. Introduction

Modeling the human brain is one of the greatest scientific challenges of our time. Computational neuroscience has made significant advancements from simulating low-level biological parts in great detail, to solving high-level problems that humans find difficult; however, we still lack a mathematical account of how biological components implement cognitive functions such as sensory processing, memory formation, reasoning, and motor control. Much work has been put into neural simulators that attempt to recreate neuroscientific data in precise detail with the thought that cognition will emerge by connecting detailed neuron models according to the statistics of biological synapses (Markram, 2006). However, cognition has not yet emerged from data-driven large scale models, and there are good reasons to think that cognition may never emerge (Eliasmith and Trujillo, 2013). At the other end of the spectrum, cognitive architectures (Anderson et al., 2004; Aisa et al., 2008) and machine learning approaches (Hinton and Salakhutdinov, 2006) have solved high-dimensional statistical problems, but do so without respecting biological constraints.

Nengo^1^ is a neural simulator based on a theoretical framework proposed by Eliasmith and Anderson (2003) called the Neural Engineering Framework (NEF). The NEF is a large-scale modeling approach that can leverage single neuron models to build neural networks with demonstrable cognitive abilities. Nengo and the NEF has been used to build increasingly sophisticated neural subsystems for the last decade [e.g., path integration (Conklin and Eliasmith, 2005), working memory (Singh and Eliasmith, 2006), list memory (Choo and Eliasmith, 2010), inductive reasoning (Rasmussen and Eliasmith, 2014), motor control (DeWolf and Eliasmith, 2011), decision making (Stewart et al., 2012)] culminating recently with Spaun, currently the world's largest functional brain model (Eliasmith et al., 2012). Spaun is a network of 2.5 million spiking neurons that can perform eight cognitive tasks including memorizing lists and inductive reasoning. It can perform any of these eight tasks at any time by being presented the appropriate series of images representing the task to be performed; for example, sequentially presenting images containing the characters *A*3[1234] instructs Spaun to memorize the list 1234. If asked to recall the memorized list, Spaun generates motor commands for a simulated arm, writing out the digits 1234. While the tasks that Spaun performs are diverse, all of the tasks use a common set of functional cortical and subcortical components. Each functional component corresponds to a brain area that has been hypothesized to perform those functions in the neuroscientific literature.

The NEF provides principles to guide the construction of a neural model that incorporates anatomical constraints, functional objectives, and dynamical systems or control theory. Constructing models from this starting point, rather than from single cell electrophysiology and connectivity statistics alone, produces simulated data that explains and predicts a wide variety of experimental results. Single cell activity (Stewart et al., 2012), response timing (Stewart and Eliasmith, 2009), behavioral errors (Choo and Eliasmith, 2010), and age-related cognitive decline (Rasmussen and Eliasmith, 2014), of NEF-designed models match physiological and psychological findings without being built specifically into the design. These results are a consequence of the need to satisfy functional objectives within anatomical and neurobiological constraints.

The transformation principle of the NEF proposes that the connection weight matrix between two neural populations can compute a non-linear function, and can be factored into two significantly smaller matrices. By using these factors instead of full connection weight matrices, NEF-designed models are more computationally efficient, which allows Nengo to run large-scale neural models on low-cost commodity hardware.

In order to make Nengo more simple, extensible, and fast, we have rewritten Nengo 2.0 from scratch in Python, leveraging NumPy (Oliphant, 2007) for manipulating large amounts of data. While NumPy is its only dependency, Nengo contains optional extensions for plotting if Matplotlib is available (Hunter, 2007) and for interactive exploration if IPython is available (Pérez and Granger, 2007). Since Nengo only depends on one third-party library, it is easy to integrate Nengo models in arbitrary CPython programs, opening up possibilities for using neurally implemented algorithms in web services, games, and other applications.

Nengo 2.0 has a simple object model, which makes it easy to document, test, and modify. Model creation and simulation are decoupled, allowing for models to be run with other simulators as drop-in replacements for Nengo 2.0's platform-independent reference simulator. To date, we have implemented one other simulator that uses PyOpenCL (Klöckner et al., 2012) to take advantage of a GPU or multicore CPU. The OpenCL simulator can simulate large models on the scale of Spaun at least 50 times faster than Nengo 1.4 using inexpensive commodity hardware.

In all, Nengo 2.0 provides a platform for simulating larger and more complex models than Spaun, and can therefore further test the NEF as a theory of neural computation.

## 2. Neural engineering framework (NEF)

The Neural Engineering Framework (NEF; Eliasmith and Anderson, 2003) proposes three quantitatively specified principles that enable the construction of large-scale neural models. Briefly, this mathematical theory defines:

1. Representation: A population of neurons collectively represents a time-varying vector of real numbers through non-linear encoding and linear decoding.
2. Transformation: Linear and non-linear functions on those vectors are computed by linear decodings that are used to analytically compute the connections between populations of neurons.
3. Dynamics: The vectors represented by neural populations can be considered state variables in a (linear or non-linear) dynamical system, and recurrent connections can be computed using principle 2.

**Figure 1:**
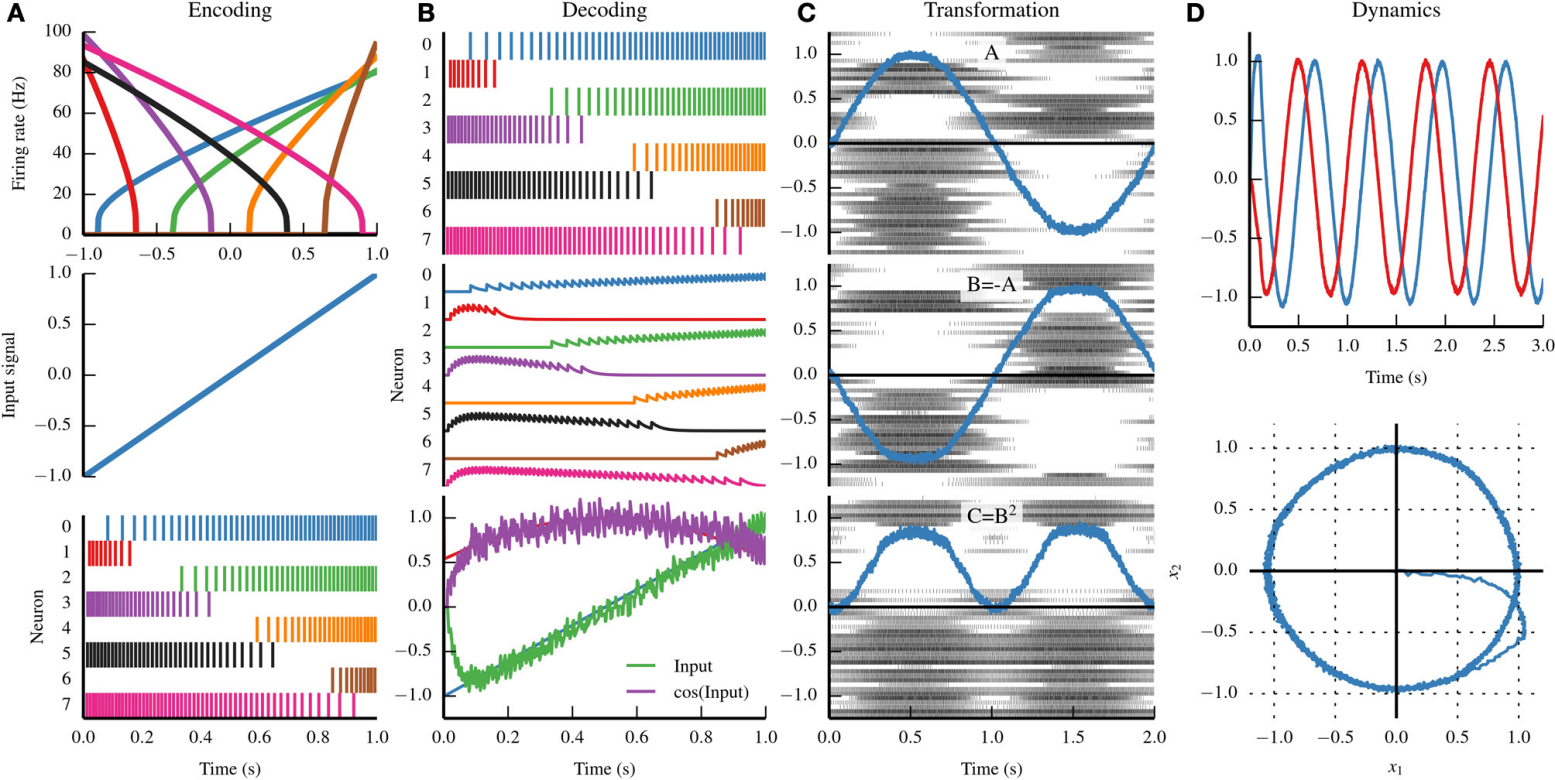
**Summary of the three principles of the Neural Engineering Framework (NEF)**. **(A)** By the representation principle, signals are encoded in neural populations based on the *tuning curve* of each neuron (top). The tuning curve describes how active a neuron is given some input signal. If we drive the eight neurons in the top panel with the input signal in the middle panel, we see the spike trains in the bottom panel. **(B)** By the representation principle, the spiking activity of a neural population can be decoded to recover the original input signal, or some transformation of that input signal. First, the firing pattern shown in the top panel is filtered with a decaying exponential filter (middle panel). The filtered activity is then summed together with a set of weights that approximates the input signal (bottom panel, green) and the cosine of the input signal (bottom panel, purple). **(C)** A sine wave is encoded by population A (top panel); the negative of that signal is projected to population B (middle panel) and the square of that signal is projected to population C (bottom panel). By the transformation principle, populations of neurons can send signals to another population by decoding the desired function from the first population and then encoding the decoded estimate into the second population. These two steps can be combined into a single step by calculating a set of weights that describe the strength of the connections between each neuron in the first population and each neuron in the second population. **(D)** A neurally implemented dynamical system has negative feedback across its two dimensions, resulting in a harmonic oscillator. The oscillator is plotted across time (top) and in state space (bottom). By the dynamics principle, signals being represented by population of neurons can be thought of as state variables in a dynamical system.

Figure 1 provides a graphical summary of these three principles.

### 2.1. Representation

Information is encoded by populations of neurons. The NEF represents information with time-varying vectors of real numbers, allowing theorists to propose possible neural computations by manipulating that information using conventional mathematics. The NEF suggests that we can characterize the *encoding* of those vectors by injecting specific amounts of current into single neuron models based on the vector being encoded. This drives the neuron, causing it to spike. With enough neurons, the originally encoded vector can be estimated through a *decoding* process. This idea is a kind of population coding (Georgopoulos et al., 1986; Salinas and Abbott, 1994), but generalized to vectors of arbitrary dimensionality.

In the encoding process, the input signal drives each neuron based on its *tuning curve*, which describes how likely that neuron is to respond to a given input signal. The tuning curve is a function of the gain of a neuron (how quickly the activity rises), the bias (the activity of a neuron given no signal), and the *encoding weight* (the direction in the input vector space that causes the neuron to be the most active). Importantly, tuning curves can be determined for any type of neuron, and therefore the encoding process (and the NEF as a whole) is not dependent on any particular neuron model.

In the decoding process, the spike trains are first filtered with an exponentially decaying filter accounting for the process of a spike generating a postsynaptic current. Those filtered spike trains are summed together with weights that are determined by solving a least-squares minimization problem. Note that these decoding weights do not necessarily depend on the input signal; instead, we typically perform this minimization on points sampled from the vector space that the population represents.

In Nengo, the representation principle can be seen in the Ensemble object (see section 3.1).

### 2.2. Transformation

Neurons communicate through unidirectional connections called synapses. When a neuron spikes, it releases neurotransmitter across the synapse, which typically causes some amount of current to be imparted in the postsynaptic (downstream) neuron. Many factors affect the amplitude of the imparted current; we summarize those factors in a scalar connection weight representing the strength of the connection between two neurons. In order to compute any function, we set the connection weights between two populations to be the product of the decoding weights for that function in the first population, the encoding weights for the downstream population, and any linear transform.

This implies that the NEF makes a hypothesis about synaptic weight matrices; specifically, that they have low rank, and can be factored into encoders, decoders, and a linear transform. Note that, in practice, we rarely use the full connection weight matrix, and instead store the encoders, decoders, and linear transform separately (i.e., the three factors of the connection weight matrix). This provides significant space and time savings during simulation.

In Nengo, the transformation principle can be seen in the Connection object (see section 3.3).

### 2.3. Dynamics

While feedforward vector transformations suffice to describe some neural systems, many require persistent activity through recurrent connections. When recurrent connections are introduced, the vectors represented by neural populations can be thought of as state variables in a dynamical system. The equations governing dynamics in such a system can be designed and analyzed using the methods of control theory, and translated into neural circuitry using the principles of representation and transformation.

In Nengo, dynamics can be seen when an Ensemble is connected to itself. Several of the Networks implemented in Nengo also exhibit dynamics.

### 2.4. NEF and nengo

Large models can be built by using the principles of the NEF as connectable components that describe neural systems, just as a circuit diagram describes an electronic circuit. The goal of Nengo is to enable modelers to create and connect those components. Ensembles describe what information is being represented, and connections describe how that information is transformed. Nengo implements those descriptions with its object model, and translates those objects to a network of interconnected neurons, situating it as a “neural compiler” that translates a high-level functional model to a low-level neural model.

## 3. Nengo object model

To describe an NEF model, Nengo defines six core objects.

1. The Ensemble contains a group of neurons that encodes a time-varying vector of real numbers.
2. The Node represents non-neural information, such as sensory inputs and motor outputs.
3. The Connection describes how nodes and ensembles are connected.
4. The Probe gathers data during a simulation for later analysis.
5. The Network encapsulates a functionally related group of interconnected nodes and ensembles.
6. The Model encapsulates a Nengo model.

These six objects contain symbolic information about a Nengo model, enabling a strict division between model construction and simulation. This allows a Nengo model to be run on multiple simulators.

### 3.1. Ensemble

An Ensemble is a population of neurons that represents information in the form of a real-valued vector. When creating an ensemble, the user must provide a name, an object that describes a population of neurons, and the dimensionality (i.e., the length of the vector it represents). For example,

nengo.Ensemble(nengo.LIF(50,
               tau_ref=0.002), 1)
describes an ensemble that uses 50 leaky integrate-and-fire neurons (Lapicque, 1907) with a 2 ms refractory period to represent a one-dimensional vector. The nengo.LIF class defines the parameters of the LIF neurons symbolically so that each simulator can compute the LIF non-linearity efficiently. The neuron model used by the ensemble is changed by passing in a different symbolic neuron object; however, the simulator used must be aware of that type of neuron.

Other attributes of the Ensemble, such as its encoding weights, can be specified either as keyword arguments to the Ensemble constructor, or by setting an attribute on the instantiated object. While an ensemble makes a hypothesis about the information being represented by neurons, these additional attributes allow modelers to set neural parameters according to *in vivo* electrophysiology data. If these attributes are not set, Nengo attempts to maintain neurobiological constraints by using default parameters consistent with neocortical pyramidal cells.

### 3.2. Node

A Node contains a user-defined Python function that directly calculates the node's outputs from its inputs at each timestep. Available inputs include the simulator timestep, the decoded output of an ensemble, or the output of another node. However, unlike ensembles, there are no constraints on the type of function that the node computes. A node can track any number of variables internally, and use the state of those variables when computing its function. For example, it can interact directly with hardware, and interface with other programs using shared memory or sockets.

Generally, a node represents information that cannot be decoded from an ensemble. As a simple example, a node can be used to model sensory stimuli that are predefined functions of time. As a more sophisticated example, a node can be used to model a complex experimental environment that both provides input to the neural model and responds to the neural model's output. Nodes allow Nengo to represent neural components, the body that those components drive, and the environment that body interacts with in a single unified model. This makes Nengo models more explicit, and enables simulators to control and optimize node execution.

### 3.3. Connection

Ensembles and nodes can be connected together in several ways. A Connection contains symbolic information about how two objects are connected. That information either includes a factored or full connection weight matrix, or includes enough information to generate weights during simulation.

When an ensemble is connected to another object, the connection implements the NEF's transformation principle. In other words, the Connection allows ensembles to project encoded information—or a transformation of that information—to another ensemble or node. This functionality is what enables Nengo models to appear conceptual, even though the underlying implementation can translate that connection to synaptic weights.

However, neurons in an ensemble can be directly connected to neurons in another ensemble with synaptic connection weights by connecting an ensemble's neurons directly to another ensemble's neurons [e.g., nengo.Connection(ens1.neurons, ens2.neurons, …)]. All connections can be temporally filtered, and the weights involved in the connection can be modified over time with learning rules.

### 3.4. Probe

A Probe monitors a particular part of another object in order to record its value throughout a simulation. Nengo models contain many variables that change over time, including membrane potentials, spike events, and encoded vectors. It is resource intensive to store the values of large numbers of variables at each timestep, and it is also not necessary, as typically only a small fraction of these variables are analyzed after a simulation. The modeler chooses which variables to record by creating a probe object.

Like nodes, a probe could be implemented outside of the neural model. However, doing so requires detailed knowledge of the simulator, and can incur significant overhead if not implemented carefully. For these reasons, we have made probes a core component of a Nengo model, and are therefore explicit and optimizable. Further, since probes are described at a symbolic level, the underlying implementation can output probed data in many different formats. Currently, simulators store probed data directly in memory, but the ability to store data in files or to stream data directly to sockets is forthcoming.

### 3.5. Network

A network is a collection of interconnected ensembles and nodes. Networks provide a way of grouping together a set of connected objects that collectively perform a complex function. Encapsulating them in a network makes its purpose explicit and hides the complexity of the function (see section 5.3 for an example). This grouping can be hierarchical networks. Network is a base class designed to be subclassed by modelers. The code that creates and connects several objects in a model can be grouped into a Network subclass with only minor changes. Nengo comes with several networks already implemented which can be used directly, or can be used as a template for modelers wanting to implement their own networks.

As a simple example, the Integrator network is composed of only one recurrently connected ensemble. By encapsulating that logic in a network, the purpose of that ensemble is made explicit. As a complex example, the BasalGanglia network is composed of five groups of ensembles connected with several specific functions that together implement a “winner-take-all” circuit. Encapsulating the code to create those ensembles and connections in a network makes a complicated section of code easy to include in many different models.

### 3.6. Model

The Model object is a container for Nengo objects. Conceptually, they are similar to networks, but are privileged in that simulators must have a model passed into their constructor, making the Model akin to a network that contains all of the objects defined for a Nengo model. A simulator's job is to simulate a model.

## 4. Nengo simulators

Decoupling model creation and simulation has been done previously by PyNN (Davison et al., 2008). In PyNN, the same Python script can be used to run a model on four different simulators. Nengo follows this programming model by decoupling neural model creation and simulation, which enables Nengo simulators to allocate memory and schedule computations in the most efficient ways possible. Simulators are given a Model as an argument; this Model is a static symbolic description. The simulator can take the model description and build whatever data structures are best suited to implement the simulation.

We have implemented a platform-independent reference simulator as an example for simulator designers. This simulator is not a specification; any object that accepts a Nengo Model as an argument is considered a Nengo simulator. To show that model creation and simulation are fully decoupled, we have also implemented an OpenCL simulator that uses PyOpenCL to parallelize computations on GPUs and multicore CPUs. However, in the remainder of this section, we will describe the reference simulator implementation; the OpenCL simulator shares many of the reference simulator's architectural choices, but the details of its implementation include OpenCL-specific optimizations that are beyond the scope of this paper.

### 4.1. Nengo reference simulator

**Figure 2:**
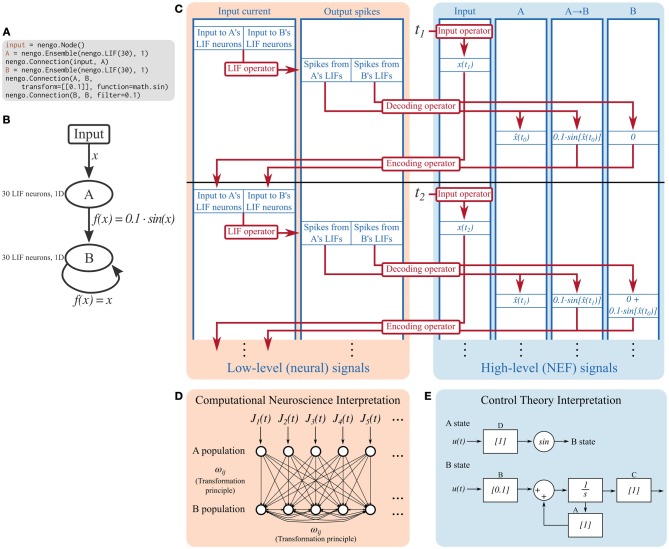
**Detailed breakdown of the Nengo reference simulator running a simple model for two timesteps**. **(A)** Code describing the model being simulated. It consists of ensemble A projecting the sine of its encoded signal to ensemble B, which is recurrently connected. **(B)** Diagram depicting the model being simulated. **(C)** A detailed diagram of how the reference simulator organizes this model. Signals (blue) represent the values tracked in the simulation. Operators (red) represent the computations done on signals. Signals can be grouped as low-level neural signals that are used to compute the non-linear functions underlying neuron models, and high-level NEF signals that are used to drive neurons and track the signals that the neurons are representing. The operators that implement the decoding and encoding steps map between the low-level neural signals and the high-level NEF signals. **(D)** The signals tracked at the low level can be interpreted as a model commonly seen in computational neuroscience literature; a population of leaky integrate-and-fire neurons is driven by some time-varying input current, *J*(*t*). These neurons project to a population of recurrently connected neurons. The connection weights between the two populations, and from the second population to itself, can be computed by the NEF's transformation principle, bypassing the need for the high-level NEF signals used by the reference simulator for speed and data collection purposes. **(E)** The signals tracked at the high level can be interpreted as a dynamical system. State variable A simply represents its input, and passes its state to a sine function which becomes the input to B. State variable B is a simple linear system that can be described with the typical *ẋ*(*t*) = *Ax*(*t*) + *Bu*(*t*) equation. These dynamical systems can be simulated directly, without the use of spiking neurons, in order to quickly analyze system behavior, if desired.

The Nengo reference simulator makes a copy of the objects in the model and fills in many of the details not specified at the symbolic level. For example, *encoders* are often not specified when the model is created, so the reference simulator randomly chooses them as unit vectors in the space that the ensemble is representing. After filling in these details, the reference simulator builds a reduced set of objects that describe the computations occurring in the model. Specifically, the simulator uses signals, which represent values, and operators, which represent computations performed on signals. Figure 2 shows the signals and operators used in a simple model.

#### 4.1.1. Signals

A Signal represents any number that will be used by the simulator. Several signals are created for each high-level Nengo object; for example, for each ensemble, the simulator creates signals that represent the high-level input signal that will be encoded to input currents, and the encoding weights. The ensemble also contains a neural population, for which the simulator creates signals that represent input currents, bias currents, membrane voltages, and refractory times for each cell.

As can be seen in Figure 2, the signals used in a Nengo simulation can be conceptually grouped into those that track low-level neural signals, and those that track high-level signals defined by the NEF. Other neural simulators only track low-level signals. Operators commonly map between related low- and high-level signals.

#### 4.1.2. Operators

Operators represent computations to be performed on signals on each timestep. Once the model has been built, only a small set of mathematical operations are necessary for simulation.

Many of the computations done in a simulation are linear transformations (e.g., the decoding and encoding steps in Figure 2), and therefore can share a common operator; this is helpful for parallelizing computations. Non-linear functions, however, require specific operators. Each supported neuron model and learning rule has an associated operator. The simulator explicitly maps from symbolic neuron objects in ensembles and from symbolic learning rule objects in connections to their associated operators.

#### 4.1.3. Reference simulator

Before the first timestep, the reference simulator

1. fills in unspecified details of high-level objects,
2. translates high-level objects to a set of signals and operators,
3. allocates NumPy arrays for each signal, and
4. sorts operators based on a dependency graph.

On each timestep, the reference simulator

1. computes each operator in order, and
2. copies probed signals to memory.

Figure 2 depicts the state of the reference simulator after two timesteps of a simple model; all subsequent timesteps perform the same operations as the first two.

## 5. Example scripts

The scripting interface provides a simple way to add Nengo objects to a model, simulate that model, and extract data collected during the simulation. Rather than list the functions in the scripting interface, we instead provide three concrete example scripts that highlight the types of models that can be built with Nengo. We have also implemented two of these three examples in PyNN to provide a comparison for the length and clarity of the code describing the models.

### 5.1. Communication channel

As detailed in section 2, NEF models are based on the principles of representation, transformation, and dynamics. One of the most important operations in a large neural model is to route represented information from one ensemble to another without any change. For example, in the visual system of Spaun, a high-dimensional representation of the visual field is compressed to a low-dimensional representation, and then sent unchanged to several areas, including the working memory and action selection networks. This routing is implemented with a transformation called a communication channel. This transform simply implements the identity function, *f*(*x*) = *x*.

**Figure 3:**
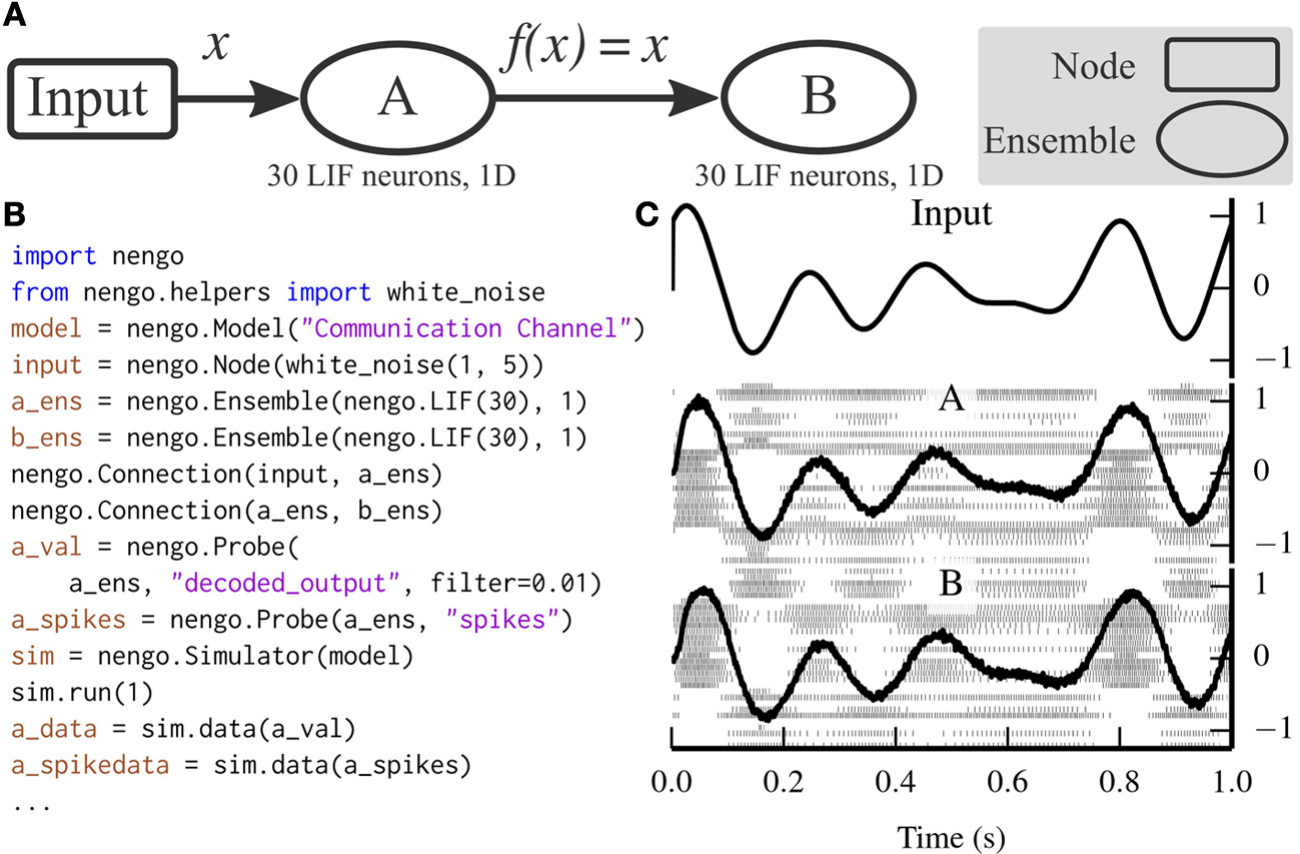
**A communication channel implemented with Nengo**. **(A)** Diagram depicting the model. Ensemble A projects its encoded signal to ensemble B unchanged. **(B)** Nengo code to build and simulate the model for 1 s. **(C)** The results of the simulation. The input signal (top panel) is white noise limited to 0–5 Hz. The signal is well-represented by both ensemble A (middle panel) and ensemble B (bottom panel) despite the neural firing patterns (underlaid in middle and bottom panels) being different.

Figure 3 depicts a scalar communication channel in which band-limited Gaussian white noise is represented in one ensemble and projected to another ensemble.

**Figure 4:**
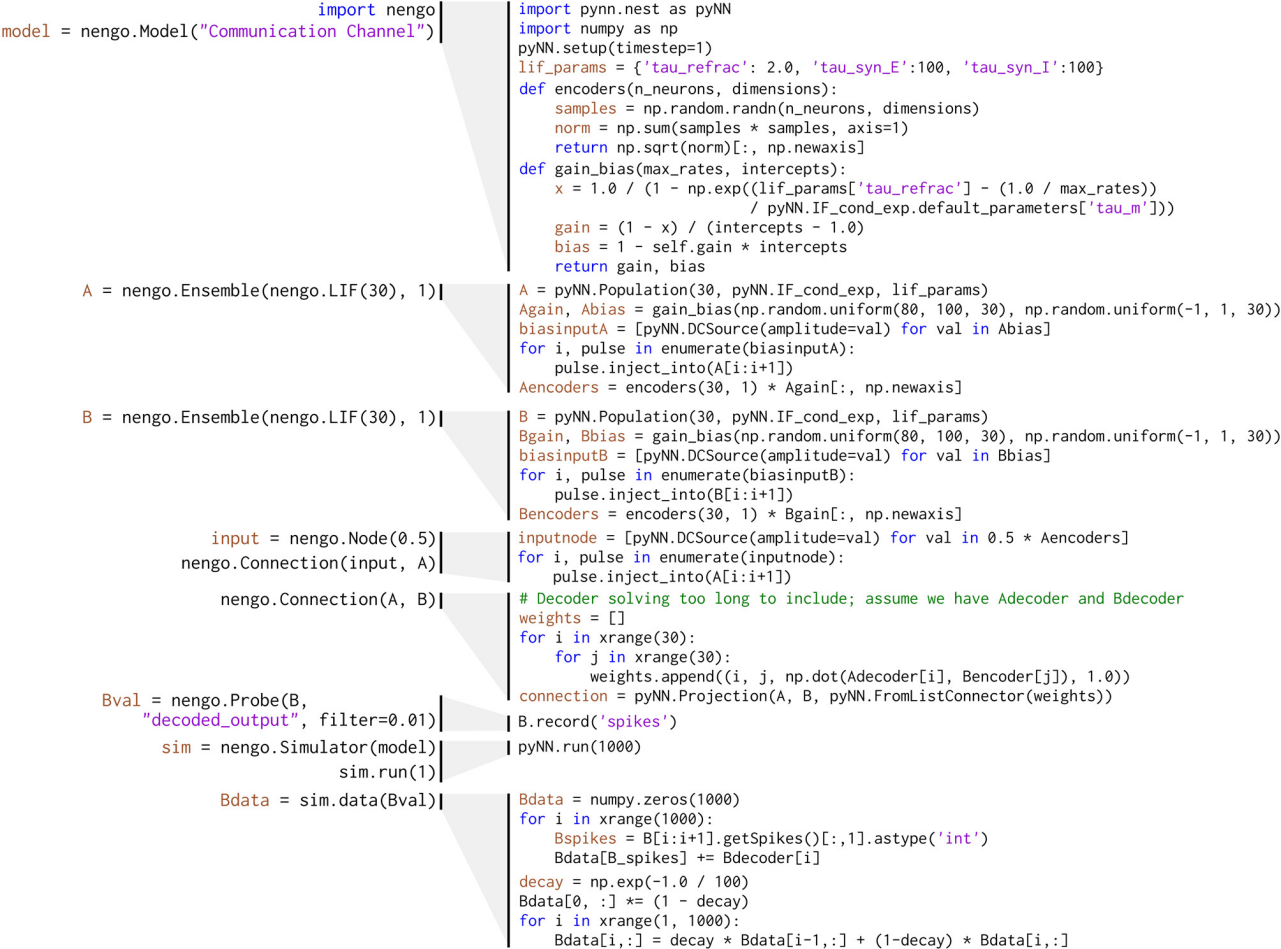
**Implementation of the communication channel (left) in Nengo and (right) in PyNN**. Solving for decoding weights takes approximately 40 lines of code, which are not included in this figure.

The communication channel is a simple enough model that it can be readily implemented in PyNN. Figure 4 compares the code for implementing a communication channel in Nengo and PyNN. This figure highlights many of the differences between Nengo models and conventional neural models; we also use these script for benchmarking (see section 6).

### 5.2. Lorenz attractor network

**Figure 5:**
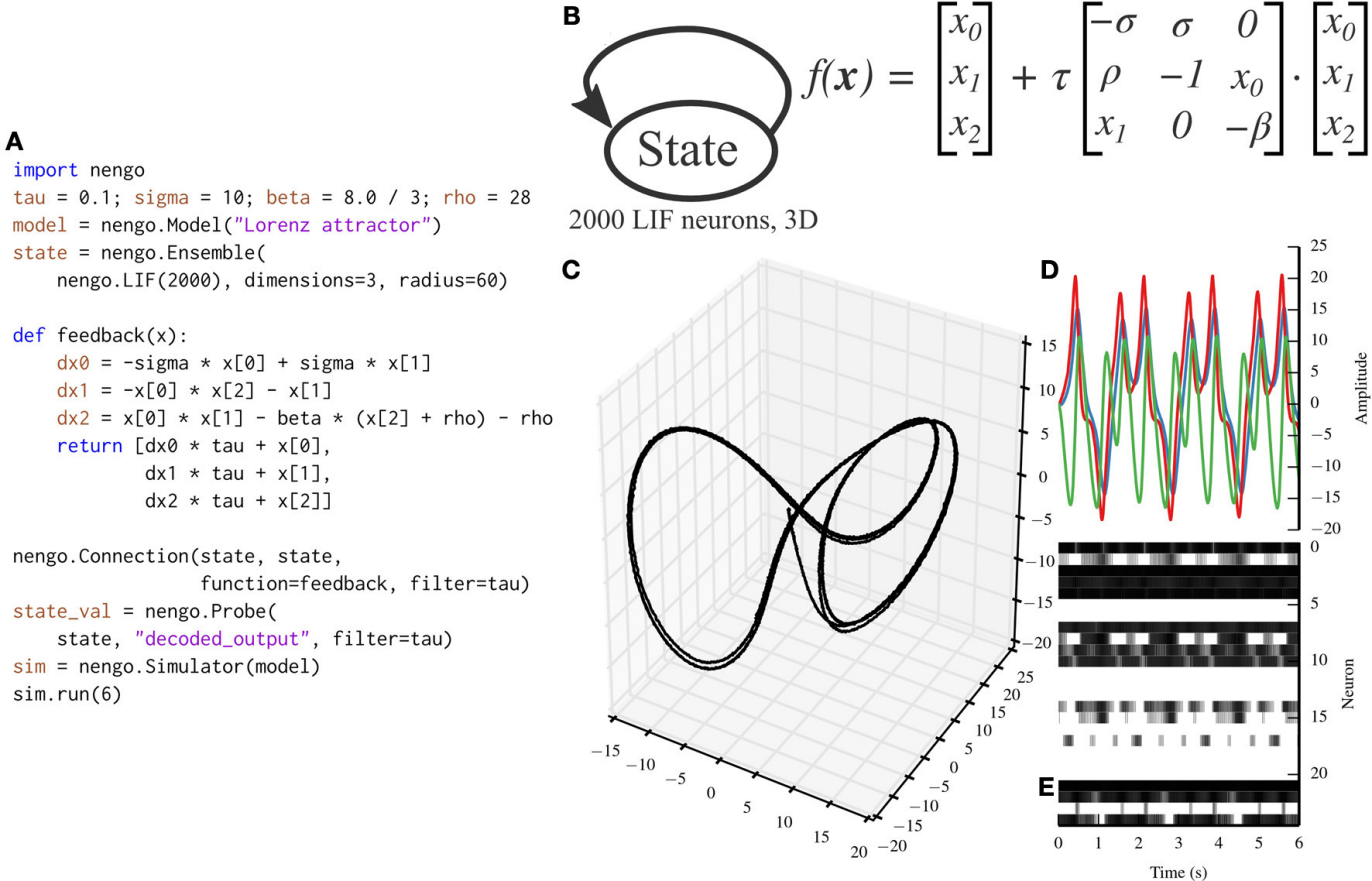
**A Lorenz attractor implemented with Nengo**. **(A)** Nengo code to build and simulate the model for 6 s. **(B)** Diagram depicting the model. The state ensemble is recurrently connected with a complex function implementing the dynamics of the Lorenz attractor. Note that this population does not receive any input that might drive its initial value; instead, the initial value is determined by the baseline firing of the 2000 leaky integrate-and-fire neurons that make up the state ensemble. **(C)** The trajectory that the state ensemble takes in its three-dimensional state space. For the parameters chosen, the trajectory takes the well-known butterfly shape. **(D)** The state vector plotted over time. **(E)** The spikes emitted by a random sample of 25 neurons from the state ensemble. Some neurons fire uniformly across the 6 s simulation, but most change depending on the state being tracked due to the recurrent connection.

While the communication channel exemplifies the representation and transformation principles of the NEF, the Lorenz attractor exemplifies the dynamics principle. Many models in theoretical neuroscience are based on attractor networks (Amit, 1992; Deco and Rolls, 2003). The NEF has been used in the past to implement many different types of attractor networks by recurrently connecting ensembles with functions that implement dynamical systems (Eliasmith, 2005). Figure 5 depicts a Nengo implementation of the Lorenz chaotic attractor with a single ensemble composed of 2000 leaky integrate-and-fire neurons. We have implemented the Lorenz attractor in PyNN for benchmarking purposes (code not shown; the PyNN script is ~100 lines long, while the Nengo script in Figure 5 is 20 lines long).

### 5.3. Circular convolution

Communication channels and attractor networks show up in many Nengo models, but are still relatively simple to implement without Nengo, as can be seen with the PyNN implementation in Figure 4. As the NEF has been used to construct larger models that have the capabilities of non-neural cognitive models, a theory called the Semantic Pointer Architecture (Eliasmith, 2013) has emerged. This theory uses high-dimensional vectors as symbol-like structures that can be combined together to form novel concepts.

**Figure 6:**
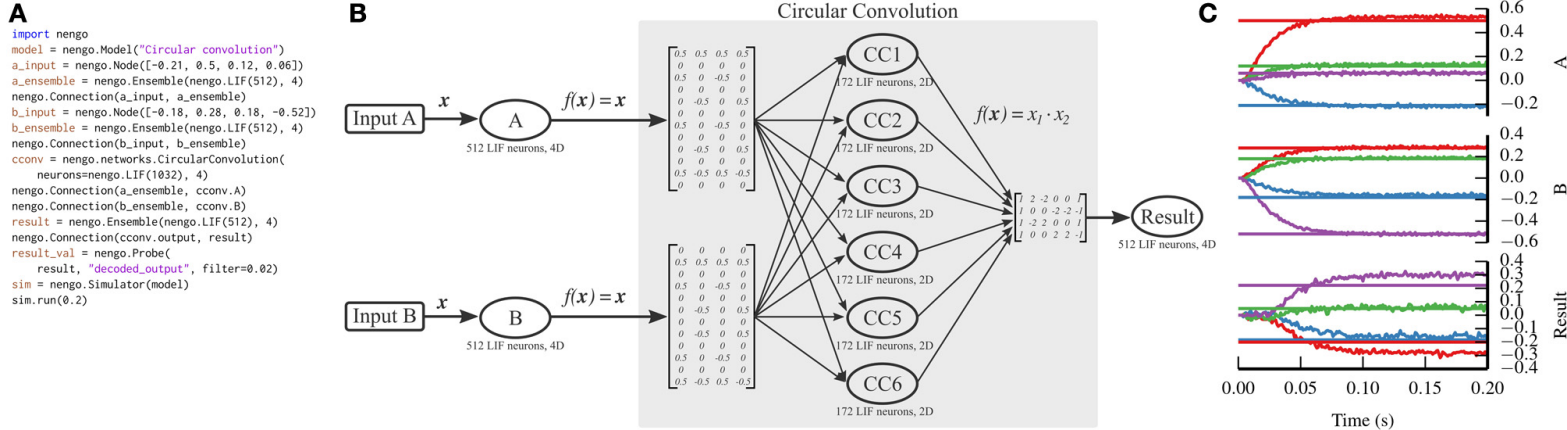
**Circular convolution implemented with Nengo**. **(A)** Nengo code to build and simulate the model for 0.2 s. **(B)** Diagram depicting the model. The input vectors, A and B, represent four-dimensional vectors which are mapped onto six ensembles within the circular convolution network through complicated transformation matrices that implement a discrete Fourier transform. Each ensemble within the network represents a two-dimensional vector. The product of the two dimensions is projected through another complicated transformation matrix that implements the inverse discrete Fourier transform, computing the final four-dimensional result. Note that the complicated parts of the model are contained within the network; the number of ensembles and the transform matrices shown are automatically generated by the network depending on the dimensionality of the input vectors. **(C)** The result of the simulation. Straight horizontal lines represent the target values that each ensemble should represent. Wavy lines represent the decoded values for each dimension represented by the A, B, and Result ensembles (top, middle, and bottom panels, respectively). The ensembles represent the correct values, after a startup transient of less than 0.1 s.

One of the functions that is performed on these vectors is to compress two *n*-dimensional vectors into a single *n*-dimensional vector, which can be decompressed into noisy versions of the two originally compressed vectors. We implement this compression using the circular convolution function. Circular convolution is best implemented in a two-layer network, rather than in a single connection, which we have simplified through the CircularConvolution network. The complexity encapsulated in that network can be seen in Figure 6.

Unlike the previous two examples, we do not implement circular convolution in PyNN. The resulting script would be too long to be instructive.

## 6. Benchmarks

While benchmark models are not indicative of performance on all models, increasing simulation speed was a primary goal of Nengo 2.0. To validate that performance has improved, we ran the models described in section 5 for various numbers of neurons and dimensions for each ensemble.

**Figure 7:**
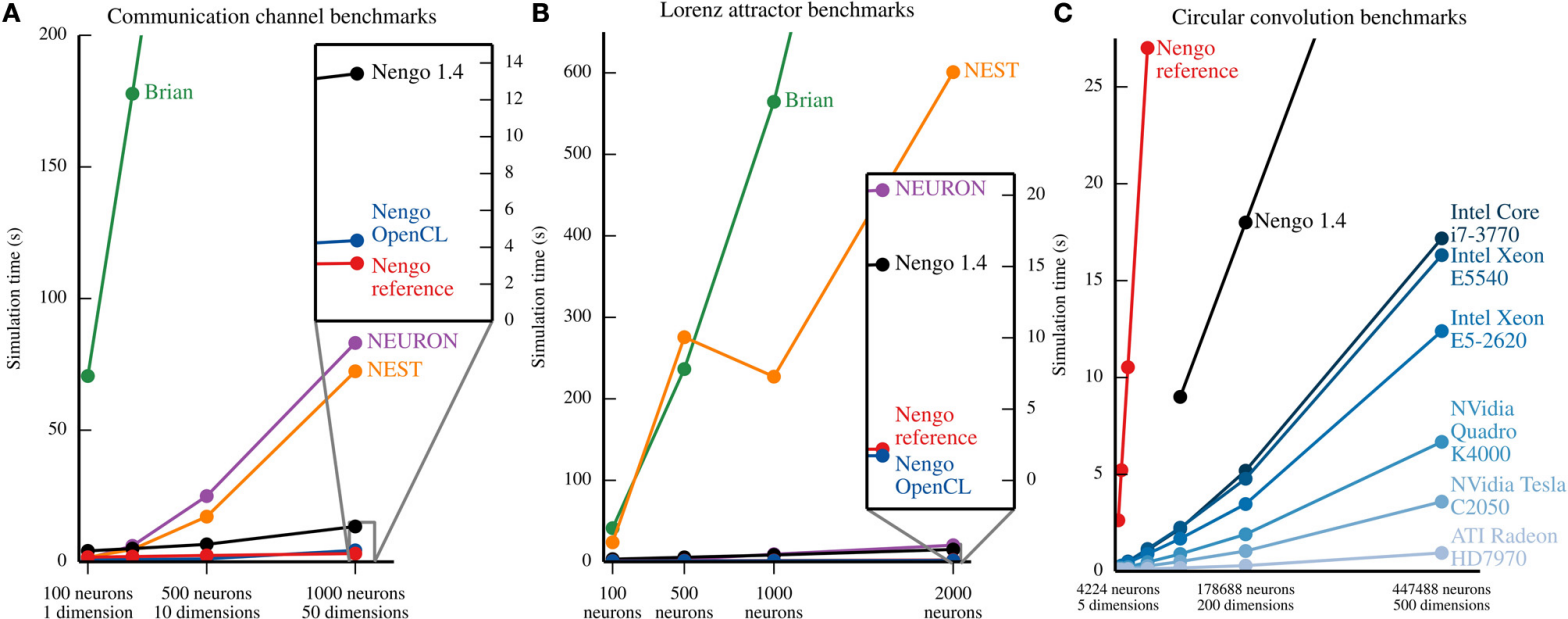
**Benchmark results for several simulators on the example models described in section 5**. In **(A)** and **(B)**, all of the simulators except the Nengo OpenCL simulator were run on an Intel Core i7-965. Nengo 1.4 used all 4 cores of this processor; all other simulator used only 1 core. The Nengo OpenCL simulator was run on an NVidia GTX280 GPU. **(A)** Benchmark results from simulating the communication channel for 10 simulated seconds at a 1 ms timestep. For all model sizes, Nengo simulators are faster than Nengo 1.4, which is significantly faster than NEURON and NEST, which are significantly faster than Brian. The full Brian results are not shown; for the largest model, the Brian simulation takes ~768 s. **(B)** Benchmark results from simulating the Lorenz attractor for 10 simulated seconds at a 1 ms timestep. For most model sizes, the results are the same as **(A)**, except that NEURON is notably faster. The full results for Brian and NEST are not shown; for the largest model, simulations in Brian and NEST take ~1467 and ~601 s, respectively. **(C)** Benchmark results from simulating circular convolution for 1 simulated second at a 1 ms timestep. For the blue lines, the simulator used was the Nengo OpenCL simulator. The CPU used for Nengo 1.4 and the Nengo reference simulator was an Intel Core i7-3770; all 4 cores were used by Nengo 1.4, while Nengo's reference simulator only used one core. For all model sizes, the OpenCL simulator is faster than the Nengo 1.4 simulator, which is faster than the Nengo reference simulator. The reference simulator was only run up to 50 dimensions. The full results for Nengo 1.4 are not shown; for the largest model, simulation with Nengo 1.4 takes ~45 s.

The communication channel and Lorenz attractor are small models that demonstrate the principles of the NEF. Their small size enables us to write PyNN scripts that implement roughly the same functionality with Brian (Goodman and Brette, 2008), NEURON (Hines et al., 2009), and NEST (Eppler et al., 2009)^2^. We ran each parameter set five times on the same machine, and plot the mean time elapsed in Figure 7. In most cases, the coefficient of variation for the five sample times is well below 0.1, except for two outliers with coefficients of 0.18 and 0.22, overall indicating that the reported means are robust. The results, shown in Figures 7A,B, suggest that all versions of Nengo are significantly faster than the simulators accessible through PyNN, especially as the size of models increases. This is likely due to Nengo's use of factorized weight matrices, rather than storing and computing with the entire weight matrix on each timestep. While NEST and NEURON were not run on multiple cores using message passing, the reference simulator of Nengo also only uses one CPU core. The results further suggest that Nengo 2.0's simulators are faster than Nengo 1.4's simulator.

As a larger-scale example, we have also benchmarked the circular convolution model. Circular convolution is an important test case, as a significant portion of Spaun's 2.5 million neurons are used to implement circular convolution. In this case, only versions of Nengo were tested. Instead of running each simulation multiple times, we instead ran the simulator for 10 timesteps in order to fill various levels of CPU or GPU cache, and then ran the simulator for 1000 more timesteps; there is very little variance using this method. As can be seen in Figure 7C, for large models, the OpenCL simulator performs much faster than Nengo 1.4; in particular, a Radeon 7970 GPU performs 500-dimensional circular convolution with about half a million neurons faster than real time, and 50 times faster than Nengo 1.4. In the 50-dimensional case, the Radeon 7970 GPU is 200 times faster than Nengo 1.4. Additionally, although both Nengo 1.4 and the OpenCL simulator on CPUs use all available CPU cores, Nengo's OpenCL simulator is many times faster.

## 7. Discussion

### 7.1. Comparison to similar projects

There are many other neural simulators dedicated to building large-scale neural models [e.g., (Goodman and Brette, 2008; Eppler et al., 2009; Hines et al., 2009)], and many tools for simulating cognitive phenomena with various levels of biologically plausibility [e.g., (Cooper and Fox, 1998; Sun, 2001; Anderson et al., 2004; Franklin et al., 2007; Aisa et al., 2008; de Kamps et al., 2008; Laird, 2012)]. However, Nengo is unique in that it is built on a theoretical framework that has enabled a cognitive architecture (the Semantic Pointer Architecture) that maintains a high level of biological plausibility, and has been validated through the Spaun model and other past work.

The most closely related projects in terms of software design are PyNN (Davison et al., 2008) and Topographica (Bednar, 2009), both of which provide a high-level scripting interface to low-level neural simulators. PyNN in particular is similar to the high-level object model in Nengo, and provides a convenient interface to the three most widely used neural simulators, according to a survey by Hanke and Halchenko (2011).

The APIs of Nengo and PyNN are similar, but differ significantly in how groups of neurons are connected together. In Nengo, connections commonly describe the mathematical operation that is performed through the connection between two ensembles; e.g., nengo.Connection(A, B, function=square) connects ensemble A to ensemble B, transmitting the square of the value represented by A to B. In PyNN, connections commonly describe features of the connection weight matrix between two populations; e.g., FixedProbabilityConnector(0.5) connects two ensembles together, with a probability of 0.5 that there will be a connection between a pair of neurons in the two populations. This difference reflects the fundamental difference that Nengo is built on a theoretical framework that enables modelers to think about information processing in the brain at a conceptual level.

On the neural simulator side, we have shown that both Nengo's reference simulator and OpenCL simulator are able to simulate two benchmark models much faster than Brian, NEST and NEURON (see Figure 7). This is, in part, because Nengo stores the factors of the connection weight matrix, rather than storing the entire matrix. However, these simulators are able to simulate many detailed neuron models and learning rules, and have access to a wealth of existing neuron models and learning rules. Because Nengo 2.0 is in an earlier development stage, many of these detailed neuron models and learning rules remain to be added. Neural simulators like Brian, NEST, and NEURON are therefore currently better suited for simulating a wider range of single cell models, while Nengo is designed for large networks of simple neural models that are connected together according to the principles of the Neural Engineering Framework, and can simulate these types of models efficiently.

One key difference between Nengo's simulators and traditional neural simulators is the target platform. While NEST and NEURON can be run on commodity hardware, networks of modest size are typically simulated in high-performance computing environments by using the Message-Passing Interface (MPI). Nengo enables large-scale neural simulation on commodity hardware, allowing researchers and hobbyists without access to large computing clusters the ability to experiment with theoretical neuroscience as is currently practiced in cutting edge research. In particular, GPUs are a powerful, low-cost computing resource that are available in most modern workstations. The OpenCL simulator makes full use of GPUs, while the previously discussed simulators currently do not.

### 7.2. Future work

Our short-term goal is to implement the Nengo 1.4 use cases not currently covered by Nengo 2.0. While we have ensured that all of the models currently used by Nengo 1.4 tutorials can be run in Nengo 2.0, several large models, like Spaun, include custom extensions written in Java. We will incorporate useful extensions in Nengo's API directly, and reimplement more specific extensions to use Nengo's API.

We are also developing two simulators that will take the same NEF model description as the existing simulators, but will target two pieces of neuromorphic hardware to achieve greater speed and power efficiency than the OpenCL simulator.

Our long-term goal is to create a graphical interface to build models and interactively inspect simulations. Nengo 1.4 includes a graphical interface that includes an interactive simulation inspector. We will use our experience building that interface to construct an improved interface for Nengo 2.0.

Additionally, we hope that in the future some work in Nengo will be done through outside contributions. Nengo 2.0 is a complete rewrite that has started with a deliberately minimal base and a well-defined API in order to make development easier than in previous versions.

## 8. Conclusion

Nengo 2.0 is the next generation of Nengo. It has been rewritten from scratch, but can already simulate most models that have been built using Nengo 1.4. It does this with 11% as many lines of code as its predecessor, and interacts seamlessly with other scientific Python tools. While the reference simulator is simple and easy to understand, the OpenCL simulator is extremely fast; it can simulate circular convolution models 50–200 times faster than Nengo 1.4, which itself is faster than alternative simulators on simpler models. This makes the creation and simulation of models that are many times larger than Spaun tractable with current hardware. These models will further test the NEF as a theory of neural computation; Nengo makes those models accessible to anyone with a modern computer.

## Author contributions

Trevor Bekolay led development of Nengo's object model and scripting interface, wrote the text of the paper, and prepared all of the figures. James Bergstra led development of the Nengo reference simulator and OpenCL simulator, edited text, created an early version of Figure 2, and ran the benchmarks shown in Figure 7C. Eric Hunsberger contributed significantly to Nengo and both of its simulators, and edited text. Travis DeWolf led development of a Theano-backed version of Nengo that identified issues with Theano, and provided the base for the version of Nengo described in this paper. Terrence C. Stewart contributed to Nengo, helped implement the PyNN scripts used in sections 5 and 6, and implemented the Nengo 1.4 scripting interface on which the Nengo object model is based. Daniel Rasmussen contributed to Nengo and the reference simulator, and edited text. Xuan Choo contributed to Nengo and the reference simulator. Aaron Russell Voelker ran the benchmarks shown in Figures 7A,B. Chris Eliasmith oversaw all development, contributed to Nengo, wrote the NEF appendix, and co-created the NEF with Charles Anderson.

## Funding

NSERC Discovery, NSERC Graduate Fellowships, NSERC Banting Fellowship, ONR (N000141310419) and AFOSR (FA8655-13-1-3084).

### Conflict of interest statement

The authors declare that the research was conducted in the absence of any commercial or financial relationships that could be construed as a potential conflict of interest.

## Footnotes

^1^Nengo is available for download through the Python Package Index at https://pypi.python.org/pypi/nengo. The full source is available at https://github.com/ctn-waterloo/nengo.

^2^We were unable to compile PCSim (Pecevski et al., 2009) on the machine on which we ran benchmarks.

## Appendix: neural engineering framework details

This description of the Neural Engineering Framework is adapted from the supplementary material of (Eliasmith et al., 2012) with permission.

The Neural Engineering Framework (NEF; Eliasmith and Anderson, 2003) provides a set of methods for building biologically plausible models based on a functional specification of a neural system. The central idea behind the NEF is that a group of spiking neurons can represent a vector space over time, and that connections between groups of neurons can compute functions on those vectors. The NEF provides a set of methods for determining what the connections need to be to compute a given function on the vector space represented by a group of neurons.

Suppose we wish to compute the function **y** = *f*(**x**), where vector space **x** is represented in population A, and vector space **y** is represented in population B. To do so, the NEF assumes that each neuron in A and B has a “preferred direction” or “encoding” vector. The preferred direction vector is the vector (i.e., direction in the vector space) for which that neuron will fire most strongly. The spiking activity of every neuron in population A can be written as

(A1) $$a_{i}(x)=G_{i}[\alpha_{i}e_{i}x+J_{i}^{\text{bias}}],$$

where *a*_*i*_ is the spike train of the *i*th neuron in the population, *G* is the spiking neural non-linearity, α is the gain of the neuron, **e** is the preferred direction (or “encoding”) vector, and *J*^bias^ is a bias current to account for background activity of the neuron. Notably, the elements in the square brackets determine the current flowing into the cell, which then drives the spiking of the chosen single cell model *G*. Equation (A1) describes how a vector space is encoded into neural spikes. This equation is depicted for a 1-dimensional vector space in Figure 1A.

The NEF proposes that linear decoders can be found to provide an appropriate estimate of any vector **x** given the neural activities from the encoding equation. We can write this as a decoding equation:

(A2) $$\hat{x}=\sum_{i}^{N}a_{i}(x)d_{i},$$

where *N* is the number of neurons in the group, **d**_*i*_ are the linear decoders, and $\hat{x}$ is the estimate of the input driving the neurons.

The NEF determines this complementary decoding for any given encoding. Specifically, this decoding is found using a least-squares optimization:

(A3) $$E=\frac{1}{2}\int {\left[x-\sum_{i}a_{i}(x)d_{i}\right]}^{2}dx,$$

where **d**_*i*_ are the decoding vectors over which this error is minimized.

In effect, this optimization process replaces learning in most other approaches to constructing neural networks. This optimization is not biologically plausible on its own, although networks generated in this manner can also be learned with a spike-based rule described in MacNeil and Eliasmith (2011).

The decoding process is depicted in Figure 1B, where the optimal linear decoders have been found and used for eight neurons. Notably, this kind of temporal decoding requires an assumption about the nature of the temporal filter being used. Here we assume that post-synaptic currents are such filters, and set the time constants to reflect the kind of neurotransmitter receptors in the connection (e.g., AMPA receptors have short time constants, ~10ms, and NMDA receptors have longer time constants, ~50 ms).

Such temporal filters map to biophysical processes once we connect groups of neurons together. Defining the encoding and decoding for groups A and B using equations (A1) and (A2) provides a means of connecting groups. For example, we can substitute the decoding of A into the encoding of B, thereby deriving connection weights

(A4) $$\omega_{ij}=d_{i}\alpha_{j}e_{j},$$

where *i* indexes the neurons in group A and *j* indexes the neurons in B. These weights will compute the function **y** = **x** (where **y** is the vector space represented in B and **x** is the vector space represented in A). For the more general case, it is possible to solve for decoders **d**^*f*^_*i*_ for any function by substituting *f*(**x**) for **x** in equation (A3), i.e., solving

(A5) $$E=\frac{1}{2}\int {\left[f(x)-\sum_{i}a_{i}(x)d_{i}^{\;f}\right]}^{2}dx.$$

In addition, if the function to be computed is linear, the relevant linear operator can be introduced into Equation (A4). The resulting general weight equation for computing any combination of linear and non-linear functions becomes:

(A6) $$\omega_{ij}=\alpha_{j}d_{i}^{\;f}Le_{j}$$

for any non-linear function *f* and *N*_*B*_ × *N*_*A*_ linear operator **L**. Computing the linear function **y** = − **x** and computing the non-linear function which is the element-wise square of the vector **x** (i.e., **y** = [*x*^2^_1_, *x*^2^_2_, …, *x*^2^_*n*_] is shown in Figure 1C.

This brief discussion is insufficient to fully introduce the generality of the NEF. However, showing how to compute linear and non-linear functions of vector spaces is sufficient for many neural computations. As these same methods can be used to compute connection weights for recurrent connections, the NEF also allows for the neural implementation of a wide variety of linear and non-linear dynamical systems in recurrent networks. A simple harmonic oscillator is shown in Figure 1D.
